# Optimization of Fermentation Conditions for the Production of the M23 Protease Pseudoalterin by Deep-Sea *Pseudoalteromonas* sp. CF6-2 with Artery Powder as an Inducer

**DOI:** 10.3390/molecules19044779

**Published:** 2014-04-16

**Authors:** Hui-Lin Zhao, Jie Yang, Xiu-Lan Chen, Hai-Nan Su, Xi-Ying Zhang, Feng Huang, Bai-Cheng Zhou, Bin-Bin Xie

**Affiliations:** 1 State Key Laboratory of Microbial Technology, Shandong University, Jinan 250100, China; E-Mails: hlzhao@yic.ac.cn (H.-L.Z.); yangjie199102@163.com (J.Y.); cxl0423@sdu.edu.cn (X.-L.C.); suhn@sdu.edu.cn (H.-N.S.); zhangxiying@sdu.edu.cn (X.-Y.Z.); fhuang@sdu.edu.cn (F.H.); 2 Marine Biotechnology Research Center, Shandong University, Jinan 250100, China; E-Mail: bczhou@qdio.ac.cn; 3 Collaborative Innovation Center of Deep Sea Biology, Shandong University, Jinan 250100, China

**Keywords:** pseudoalterin, fermentation, response surfaces methodology, M23 proteases, deep-sea bacterium

## Abstract

Proteases in the M23 family have speciﬁc activities toward elastin and bacterial peptidoglycan. The peptidoglycan-degrading property makes these proteases have potential as novel antimicrobials. Because M23 proteases cannot be maturely expressed in *Escherichia coli*, it is significant to improve the production of these enzymes in their wild strains. Pseudoalterin is a new M23 protease secreted by the deep-sea bacterium *Pseudoalteromonas* sp. CF6-2. In this study, the fermentation conditions of strain CF6-2 for pseudoalterin production were optimized using single factor experiments and response surface methodology to improve the enzyme yield. To reduce the fermentation cost, bovine artery powder instead of elastin was determined as a cheap and efficient inducer. Based on single factor experiments, artery powder content, culture temperature and culture time were determined as the main factors influencing pseudoalterin production and were further optimized by the central composite design. The optimal values of these factors were determined as: artery powder of 1.2%, culture temperature of 20.17 °C and culture time of 28.04 h. Under the optimized conditions, pseudoalterin production reached 100.02 ± 9.0 U/mL, more than twice of that before optimization. These results lay a good foundation for developing the biotechnological potential of pseudoalterin.

## 1. Introduction

Proteases in the M23 family defined in the MEROPS database [1] have a preference for glycyl bond cleavage [2,3,4,5,6,7]. Because of the abundance of glycine residues in tropoelastin sequences [8], elastin is a good substrate for proteases in family M23 [1]. In addition, M23 proteases are also known as bacteriocins that are speciﬁcally capable of cleaving the cross-linking pentaglycine bridges in peptidoglycans in bacterial cell walls [2,9], which makes these enzymes have potential as novel antimicrobials. The efficacy of two M23 bacteriocins, staphylolysin and lysostaphin, on *Staphylococcus aureus* keratitis treatment in animal models has been determined [10,11,12]. In the treatment of endophthalmitis in rat and rabbit models, staphylolysin could control the growth and reproduction of *S. aureus* and significantly reduced the quantity of bacteria in inner cornea. Compared with vancomycin, staphylolysin had a more rapid efficacy and was nontoxic for the animal subjects [10,11]. Lysostaphin secreted by *Streptococcus simulans* not only had an absolute cracker advantage on *S. aureus*, but also had lysis activities to methicillin-resistance *S. aureus* (MRSA) and vancomycin-intermediate *S. aureus* (VISA) [12]. These investigations suggest that proteases in the M23 family have a good prospect on the clinical treatment of antibiotic resistant infections.

Pseudoalterin is a new member of the M23 family, which is the most abundant extracellular protease secreted by *Pseudoalteromonas* sp. CF6-2 (hereafter called CF6-2) isolated from the deep-sea sediment of the South China Sea at a water depth of 2441 m [13]. Compared to other M23 proteases, pseudoalterin could degrade insoluble elastins at a much higher speed because it has a distinct elastolytic mechanism. Different from the reported mechanism that the M23 proteases only cleave glycyl bonds in elastin, pseudoalterin cleaves both the glycyl bonds in the hydrophobic regions and the peptide bonds in the hydrophilic regions in elastin [14]. This broad specificity implies that pseudoalterin may have high efficacy on bacterial cell wall lysis and merits further study and development.

Because M23 proteases have promising biotechnological potentials, improving production and reducing production costs are necessary before they are developed for application. Heterogenous expression such as in *Escherichia* *coli* is now the common way to improve enzyme production and reduce production cost. However, because M23 proteases are not autoprocessed to a mature form [1], it is almost impossible to express them in a mature form in *E. coli*. For this reason, it is significant to improve the production of these enzymes in their wild strains and to simultaneously reduce production costs.

Like other M23 proteases, mature pseudoalterin cannot be expressed in *E.* *coli*. The aim of this study is to lower fermentation cost and to increase pseudoalterin production by optimizing the fermentation conditions of CF6-2. Pseudoalterin is an induced enzyme, and elastin was found to be a good inducer for pseudoalterin production [14]. Because elastin is too expensive as a pseudoalterin inducer in CF6-2 fermentation, various protein- or elastin-containing materials were tested as pseudoalterin inducer candidates in an attempt to lower fermentation cost in this study. Bovine artery powder was chosen as the pseudoalterin inducer instead of elastin and response surface methodology (RSM) was further employed to optimize the fermentation conditions for pseudoalterin production. Finally, pseudoalterin production was increased by more than two folds, which is beneficial for further study on the biotechnological potential of pseudoalterin.

## 2. Results and Discussion

### 2.1. Effect of Different Inducers on Pseudoalterin Production

Inducer is one of the most important factors affecting protease production. A wide variety of purified animal proteins and crude plant tissues, including casein, gelatin, elastin, soybean meal, corn powder and wheat bran, have been used in protease production [13,14,15]. The results showed that the pseudoalterin production of CF6-2 varied with these inducers (Table 1). When bovine artery (wet, 2.5%) was used as an inducer, CF6-2 displayed the maximum pseudoalterin production (54.6 ± 2.4 U/mL), which was even higher than that induced by 0.3% of elastin powder (46.9 ± 3.9 U/mL). Other animal tissues or extracts led to moderate pseudoalterin production, while the other nitrogen sources had little inducing effect. Based on these data, bovine artery was used in the RSM optimization experiments. As far as we know, artery has never been reported as a protease inducer before.

**Table 1:** Effects of various inducers on pseudoalterin production of CF6-2 *^a^*.

Inducers	Elastolytic activity after different fermentation time (U/mL)					
	24 h	36 h	48 h	60 h	72 h	84 h
Elastin (0.3%)	16.5 ± 0.7	32.4 ± 4.0	35.3 ± 3.5	42.9 ± 3.2	46.9 ± 3.9	41.4 ± 3.6
Artery (wet, 2.5%)	12.6 ± 0.8	27.5 ± 1.0	42.1 ± 2.0	45.8 ± 1.6	54.6 ± 2.4	42.7 ± 1.7
Bovine lung (wet, 2.5%)	6.2 ± 0.8	9.2 ± 1.7	7.1 ± 1.2	7.2 ± 1.3	14.5 ± 1.4	11.4 ± 0.6
Ligament (wet, 2.5%)	10.3 ± 0.4	18.0 ± 0.9	11.9 ± 0.6	10.0 ± 0.7	18.1 ± 0.7	16.2 ± 1.2
Chicken intestine (wet, 2.5%)	6.7 ± 1.2	9.7 ± 1.0	8.3 ± 0.4	7.2 ± 0.4	14.9 ± 1.0	11.1 ± 1.0
Beef extract (1%)	4.8 ± 0.5	11.6 ± 0.4	11.2 ± 0.0	14.2 ± 0.8	13.1 ± 0.9	12.9 ± 1.3
Gelatin (1%)	ND *^b^*	4.3 ± 0.7	1.1 ± 0.0	0.9 ± 0.2	ND *^b^*	ND *^b^*
Yeast extract peptone dextrose (1%)	ND *^b^*	3.6 ± 0.2	2.2 ± 0.1	3.8 ± 0.2	2.4 ± 0.2	2.2 ± 0.2
BSA (1%)	1.1 ± 0.0	1.0 ± 0.0	1.3 ± 0.1	1.0 ± 0.0	ND *^b^*	ND *^b^*
Peptone (1%)	ND *^b^*	1.6 ± 0.1	1.3 ± 0.1	3.1 ± 0.2	1.1 ± 0.1	2.0 ± 0.0
Soybean meal (1%)	ND *^b^*	ND *^b^*	0.9 ± 0.0	1.8 ± 0.4	1.4 ± 0.4	2.2 ± 0.2
Casein (1%)	ND *^b^*	3.6 ± 0.3	3.1 ± 0.5	5.0 ± 0.1	1.4 ± 0.0	2.9 ± 0.2

*^a^* The data expressed as means ± standard were calculated from three replicates. *^b^* Not detectable.

### 2.2. Single Factor Experiments

**Figure 1:**
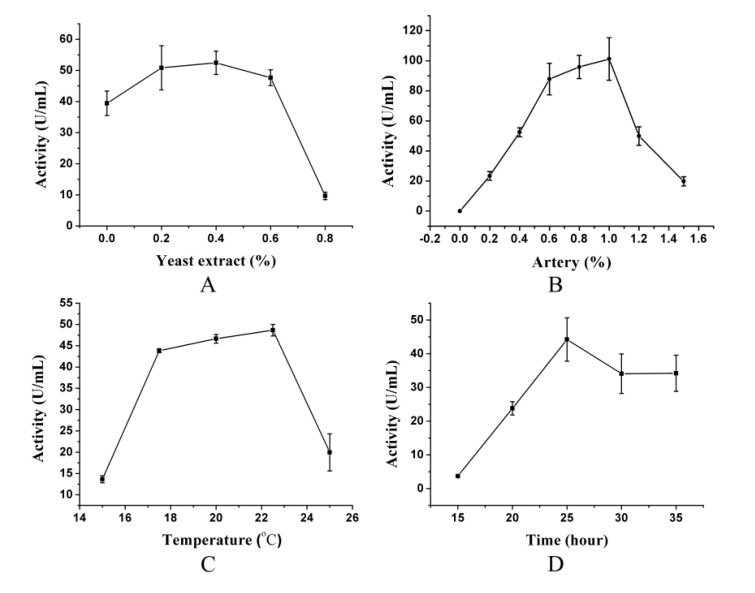
Effects of four factors on the pseudoalterin production of CF6-2. The four tested factors are yeast extract content (**A**) and artery powder content (**B**) in the medium, culture temperature (**C**) and culture time (**D**). CF6-2 was cultured under the fermentation conditions described in Experimental Section with a tested single factor being changed. The graph shows data from triplicate treatments (mean ± S.D.).

After artery powder was determined as an inducer, single factor experiments were performed to investigate the effect of four factors (the concentration of nitrogen resource, the content of inducer, the culture temperature and the culture time) on pseudoalterin production. As shown in Figure 1A, addition of 0.2%–0.6% yeast extract in the medium caused only a little increase in the production of pseudoalterin. When the content of yeast extract was further increased, the production of pseudoalterin declined dramatically, probably because more than 0.6% yeast extract in the medium could meet the nutritional needs of CF6-2. It was also noted that, even when no yeast extract was added, the pseudoalterin production was 39.44 ± 3.93 U/mL, which is 80% of the maximum production (52.44 ± 3.76 U/mL). Therefore, though low content of yeast extract (≤0.6%) is important for the production of pseudoalterin, variance of content in the range of 0~0.6% has only a little effect on the optimization of pseudoalterin production. To simplify the further optimization, the yeast extract content was set as a constant and a content of 0.3% was used to balance the cost and effect. The effect of artery powder content in the range of 0%–1.5% was also tested. Pseudoalterin production increased significantly with artery powder content, and came to a head at 1.0% (Figure 1B). Culture temperature also affected pseudoalterin production. Our results showed that the optimal temperature range for pseudoalterin production of CF6-2 was 17.5–22.5 °C (Figure 1C). Figure 1D shows the change of pseudoalterin production with culture time, which indicated that pseudoalterin production reached the maximum when CF6-2 was cultured for 25 h in the basal medium at 15 °C. Based on these results, the artery powder content, the culture temperature and culture time were chosen for further optimization.

### 2.3. Central Composite Design (CCD) and Response Surface Analysis

Among various methods used for optimization of culture conditions and medium composition, RSM is the most effective method used in improving microbial enzymes production [16,17,18], and CCD is often employed for optimization of multiple variables to estimate the relationship between the variables and responses [19]. Based on single factor experiments, three variables significantly influencing pseudoalterin production were chosen for further optimization, including artery powder content (X1), culture temperature (X2) and culture time (X3). In order to study the effect of their interactions on the pseudoalterin production (Y), CCD was performed ranging from −1.68 to +1.68 in relation to pseudoalterin production. The design matrix and the corresponding measured results are shown in Table 2. Analysis of the experimental data using multiple regression (Table 3) indicated that the independent variables and the dependent variables are related by the following second-order polynomial equation:

Y (U/mL) = −1171.57 + 191.75*X*_1_ + 93.34*X*_2_ + 16.37*X*_3_ − 0.55*X*_1_*X*_2_ + 2.07*X*_1_*X*_3_ + 0.17*X*_2_*X*_3_ − 108.41*X*_1_^2^ − 2.42*X*_2_^2^ − 0.40*X*_3_^2^ (1)

The fit of the model was checked by the coefficient of determination *R*^2^, which was 0.9742, indicating that 97.42% of the variability in the response could be explained by the model. The statistical significance of the quadratic regression equation was evaluated with the F-test analysis. The “Model *F*-value” was 41.99, indicating that the model was significant and that there was only a 0.01% chance that a “Model *F*-Value” could occur due to noise (*p* < 0.0001). Moreover, the *p*-value of the lack of fit was 0.5947, indicating that the Lack of Fit was not significant relative to the pure error.

**Table 2:** The matrix of the CCD experiment and the corresponding experimental data.

Run	X1 (artery)		X2 (temperature)		X3 (time)		Activity (U/mL)
	Coded level	Real level (%)	Coded level	Real level (°C)	Coded level	Real Level (h)	
1	0	0.9	+1.68	24.0	0	25.0	54.38
2	0	0.9	0	19.0	+1.68	33.4	75.99
3	+1	1.2	+1	22.0	−1	20.0	66.51
4	+1	1.2	+1	22.0	+1	30.0	99.23
5	−1	0.6	+1	22.0	+1	30.0	63.37
6	0	0.9	0	19.0	0	25.0	89.15
7	0	0.9	0	19.0	−1.68	16.6	52.76
8	−1	0.6	−1	16.0	+1	30.0	26.42
9	0	0.9	−1.68	14.0	0	25.0	7.79
10	0	0.9	0	19.0	0	25.0	91.40
11	0	0.9	0	19.0	0	25.0	89.83
12	+1	1.2	−1	16.0	+1	30.0	56.65
13	+1.68	1.4	0	19.0	0	25.0	78.99
14	−1	0.6	+1	22.0	−1	20.0	50.66
15	+1	1.2	−1	16.0	−1	20.0	41.81
16	0	0.9	0	19.0	0	25.0	93.82
17	−1	0.6	−1	16.0	−1	20.0	16.37
18	−1.68	0.4	0	19.0	0	25.0	50.78
19	0	0.9	0	19.0	0	25.0	90.16
20	0	0.9	0	19.0	0	25.0	106.42

**Table 3:** Variance analysis of response surface quadratic model for elastolytic activity of the fermented broth of CF6-2.

Source	df	Elastolytic activity of fermented broth *^a^*			
		Sum of Squares	Mean Square	*F* Value	*p*-value Prob > *F*
Model	9	14610.93	1623.44	41.99	<0.0001 *^b^*
*X*_1_	1	1755.06	1755.06	45.39	<0.0001 *^b^*
*X*_2_	1	3455.08	3455.08	89.36	<0.0001 *^b^*
*X*_3_	1	876.13	876.13	22.66	0.0008 *^b^*
*X*_1_*X*_2_	1	1.97	1.97	0.051	0.8258
*X*_1_*X*_3_	1	76.86	76.86	1.99	0.1889
*X*_2_*X*_3_	1	52.68	52.68	1.36	0.2702
*X*_1_^2^	1	1371.88	1371.88	35.48	0.0001 *^b^*
*X*_2_^2^	1	6815.04	6815.04	176.25	<0.0001 *^b^*
*X*_3_^2^	1	1423.06	1423.06	36.80	0.0001 *^b^*
Residual	10	386.67	38.67		
Lack of Fit	5	171.62	34.32	0.80	0.5947
Pure Error	5	215.05	43.01		
Cor Total	19	14997.60			

*^a^* R^2^ = 0.9742; Adj R^2^ = 0.9510 CV = 9.55%. *^b^* Model terms are significant.

The coefficient of variation (CV) indicates the ratio of the standard error of estimate to the mean value of the observed response. Generally, a model can be considered reasonably reproducible if the CV is not greater than 10% [20]. Here, the CV value was 9.55%, indicating a high degree of precision in the experiment. All these parameters showed a good agreement between the experimental and predicted values and implied that the model was suitable for the simulation of pseudoalterin production of CF6-2.

**Figure 2:**
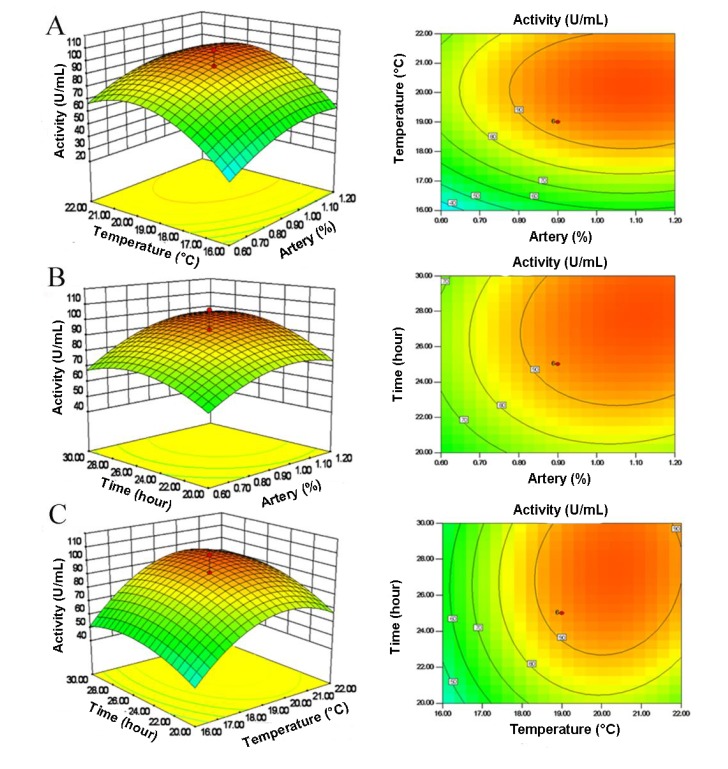
Three-dimensional plots (left) and corresponding contour plots (right) of the effect of three variables on pseudoalterin production. When the effect of two variables was plotted, the other variable was set at the central level. (**A**) interaction between artery and culture temperature; (**B**) interaction between artery and culture time; (**C**) interaction between culture temperature and culture time.

The three-dimensional response surfaces and their two-dimensional contour plots depict the interactions between the two variables by keeping another variable at its zero level (Figure 2). It has been reported that elliptical contours mean perfect interactions between the independent variables [21,22]. As shown in Figure 2, the shapes of the three contour plots are all elliptical, indicating that the mutual interactions between every two of the three variables were significant. Elastase activity of the fermented broth varied significantly upon changes in artery powder content, culture temperature or culture time. By solving the inverse matrix using Expert-Design software, the model predicted a maximum pseudoalterin production of 101.385 U/mL, under the optimal values of three variables: 1.2% artery powder, 20.17 °C and 28.04 h.

### 2.4. Verification of Optimal Conditions

**Figure 3:**
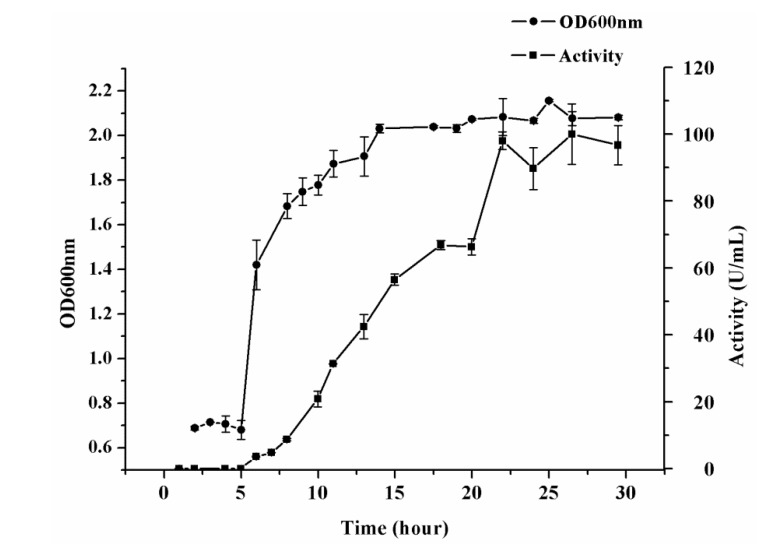
The growth and pseudoalterin production of strain CF6-2 cultured under the predicted optimal conditions. The culture medium consisted of 1.2% artery, 0.3% yeast extract, 0.5 mM CaCl_2_ and 0.5 mM Na_2_HPO_4_, the initial pH was adjusted to 8.5, the culture temperature was 20 °C, and the culture volume was 50 mL/500 mL. The graph shows data from triplicate treatments (mean ± S.D.).

To validate the optimization results, CF6-2 was cultured under the following conditions: the medium contained 0.3% (w/v) yeast extract, 0.5 mM CaCl_2_, 0.5 mM Na_2_HPO_4_, 1.2% (w/v) artery powder, 3% (w/v) sea salt; the initial pH was 8.5; the culture temperature was 20°C; the culture time was 28 h. Under these conditions, CF6-2 reached the stationary phase after 15 h (Figure 3), and pseudoalterin production reached the maximum of 100.02 ± 9.0 U/mL at 26.5 h (Figure 3), which was almost equal to the predicted value (101.385 U/mL). The excellent agreement between the predicted and measured values indicates the model validation and existence of an optimal point.

## 3. Experimental Section

### 3.1. Strain and Media

*Pseudoalteromonas* sp. CF6-2 was isolated from a deep-sea sediment in the South China Sea at a water depth of 2441 m at site 119°30.060' E, 22°0.316' N during the South China Sea Open Cruise of R/V Shiyan 3 [13]. The strain CF6-2 was cryopreserved at −80 °C supplemented with 12% glycerol. At the same time, it was routinely cultured at 15 °C for 2 d on a plate containing 10 g/L peptone, 5 g/L yeast extract, artificial sea water and 15 g/L agar (pH 8.5), which was then stored at 4 °C for short‑term use. The basal medium for pseudoalterin production of CF6-2 contained 0.2% yeast extract (w/v), 0.3% bovine elastin (Sigma, Ronkonkoma, NY, USA), 0.5 mM CaCl_2_, 0.5 mM Na_2_HPO_4_ and artificial sea water; the initial pH of the medium was adjusted to 8.5. All chemicals used in this study were of analytical reagent grade. Bovine artery was purchased from a local slaughter house. Artery powder was prepared as follows: fat was carefully removed from the vessel wall, and the residual artery tissue was cut into pieces, lyophilized, grinded and sieved by using a 20 mesh sieve.

### 3.2. Inoculum Preparation and Flask Fermentation

For inoculum preparation, CF6-2 was inoculated into a marine broth medium composed of 10 g/L peptone, 5 g/L yeast extract and artificial sea water (pH 8.5), and incubated at 15 °C, 200 rpm for 24 h. For fermentation, 1% (v/v) inoculum was inoculated into 50 mL basal medium in a 500 mL Erlenmeyer flask, which was then incubated at 15 °C, 200 rpm for 48 h.

### 3.3. Determination of Elastase Activity

Fermentation culture of CF6-2 was centrifuged at 11,000 rpm, 4 °C for 15 min, and the supernatant was used as enzyme solution in elastase activity assay after appropriately diluted with 50 mM Tris-HCl (pH 9.0). A mixture of 5 mg elastinorcein and 0.25 mL enzyme solution was incubated at 25 °C for 1 h with continuous shaking. The residual elastinorcein was removed by centrifugation and the absorption at 590 nm of the supernatant was measured. One unit of enzyme activity (U/mL) was defined as the amount of enzyme that caused an increase of 0.01 U of absorbance at 590 nm per min [15].

### 3.4. Effect of Different Inducers on Pseudoalterin Production

Bovine elastin (0.3%) in the basal medium was replaced by 2.5% (w/v) wet animal tissues, namely chicken intestine, bovine ligament, bovine lung or bovine artery, or 1% other nitrogen sources, including yeast extract peptone dextrose, gelatin, BSA, casein, peptone, beef extract, and soybean meal. Strain CF6-2 was cultured in each modified medium under the fermentation conditions described above in triplicate. The results were expressed as means ± standard deviation.

### 3.5. Optimization by Single Factor Experiments

Four single factors were detected for their influences on the production of pseudoalterin, including the yeast extract concentration of the medium (0, 0.2%, 0.4%, 0.6% or 0.8%), the concentration of bovine artery powder (0, 0.2%, 0.4%, 0.6%, 0.8%, 1.0%, 1.2% or 1.5%), culture temperature (15, 17.5, 20, 22.5 or 25 °C) and culture time (15 h, 20 h, 25 h, 30 h or 35 h). CF6-2 was cultured under the fermentation conditions described above with a tested single factor being changed. All treatments were performed in triplicate and expressed as means ± standard deviation.

### 3.6. Optimization by Response Surface Methodology (RSM)

The concentration of bovine artery powder, culture temperature and culture time were further optimized by CCD of RSM. The range and center point values of the three independent variables were set based on the results from single factor experiments. For these factors, the trial was a 23 factorial design with six axial points (a = 1.68) and six replicates of the center points, resulting in a total of 20 experiments. The elastase activity of CF6-2 fermentation broth was taken as the response value for the combination of three variables given in Table 2. Experimental runs were randomized to estimate a pure error sum of squares in the observed responses. The content of yeast extract, CaCl_2_, Na_2_HPO_4_ in the medium were set at 0.3%, 0.5 mM, 0.5 mM, respectively, and the initial pH was 8.5.

### 3.7. Statistical Analysis

The results of CCD were expressed as the following second-order polynomial regression model (2):

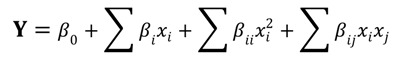
(2)


where Y is the predicted response, β_0_ is the center point of the system, β_i_ is the linear coefficient, β_ii_ is the quadratic coefficient, β_ij_ is the interactive coefficient, while x_i_, x_i_^2^ and x_j_ are the linear, quadratic and interaction terms of the independent variables, respectively [23]. The final model was expressed as surface and contour plots to reveal the relationship between each independent variable and the response [24]. Experimental designs and the analysis of polynomial coefficients were carried out using a trial version of Design-Expert software [25]. Statistical analysis of the model was performed to evaluate the analysis of variance (ANOVA).

## 4. Conclusions

The results in this study indicate that single factor experiments and CCD are an efficient and feasible approach for optimization of CF6-2 fermentation conditions to improve the production of pseudoalterin. A maximum pseudoalterin yield of 100.02 ± 9.0 U/mL was achieved under the optimized conditions, whereby CF6-2 was cultured at 20.17 °C for 28.04 h in a medium containing 1.2% artery powder content, 0.3% yeast extract, 0.5 mM CaCl_2_, 0.5 mM NaH_2_PO_4_ with an initial pH value of 8.5 and a medium volume of 10%. This yield is more than twice of that before optimization (46.9 ± 3.9 U/mL). Validation experiments also verify the accuracy of the model, which shows that the predicted value agrees well with the experimental values. In addition, the fermentation cost is also reduced due to the use of artery powder instead of elastin product. Our results provide a basis for further study on large scale fermentation of CF6-2 for pseudoalterin production.
